# Face coverings for the public: Laying straw men to rest

**DOI:** 10.1111/jep.13415

**Published:** 2020-05-26

**Authors:** Trisha Greenhalgh

**Affiliations:** ^1^ Nuffield Department of Primary Care Health Sciences University of Oxford Oxford UK

**Keywords:** evidence‐based medicine, public health, systematic reviews

## Abstract

Background

This article responds to one by Graham Martin and colleagues, who offered a critique of my previous publications on face coverings for the lay public in the Covid‐19 pandemic. Their paper reflects criticisms that have been made of face coverings policies more generally.

Method

Narrative rebuttal.

Results

I address charges that my coauthors and I had misapplied the precautionary principle; drawn conclusions that were not supported by empirical research; and failed to take account of potential harms

But before that, I remind my critics that the evidence on face coverings goes beyond the contested trials and observational studies they place centre stage. I set out some key findings from basic science, epidemiology, mathematical modelling, case studies, and natural experiments, and use this rich and diverse body of evidence as the backdrop for my rebuttal of their narrowly framed objections. I challenge my critics' apparent assumption that a particular kind of systematic review should be valorised over narrative and real‐world evidence, since stories are crucial to both our scientific understanding and our moral imagination.

Conclusion

I conclude by thanking my academic adversaries for the intellectual sparring match, but exhort them to remember our professional accountability to a society in crisis. It is time to lay straw men to rest and embrace the full range of evidence in the context of the perilous threat the world is now facing.

## 1 INTRODUCTION

Since the Covid‐19 pandemic emerged, I have coauthored several articles in both the academic and lay literature supporting the wearing of face coverings and masks by the general public.^1^, ^2^, ^3^ In response to negative criticism on social media, I put out a challenge: either write a point by point critique of my papers or back off. Martin et al responded with a preprint paper^4^ and a rapid BMJ response.^5^ The points made in their publications reflect those of other critics of face coverings as a policy response to Covid‐19.

Later in this article, I will address the substantive scientific points made by Martin et al, which I will put in italics. I have tried to represent their points faithfully, without exaggeration, and apologize in advance if I have not captured their intended nuances. But first, let me highlight a subtle rhetorical move by Martin et al: *they completely ignore various types of evidence*—including basic science, mathematical modelling and real‐world case examples of asymptomatic transmission and super‐spreader events. Before addressing what they did talk about in their paper,^4^ I set out some important scientific evidence that they did *not* talk about. I draw heavily on the primary sources cited in the narrative review by Howard et al.^6^ I deliberately avoid the term “mask” when referring to a cloth face covering (either home‐made or purchased) used by a member of the public.

## 2 A WIDER EVIDENCE BASE

The basic science of Covid‐19 is important. The Sars‐CoV‐2 virus which causes this disease replicates in the upper respiratory tract (in contrast to the causative agent of Sars‐CoV‐1, which is a less contagious lower respiratory tract virus).^7^, ^8^ This means it is likely to be transmitted mainly by droplets (which is why there is so much emphasis on hand‐washing, since droplets contaminate surfaces).^9^ Droplets emitted from the human respiratory tract (which are relatively large, and are emitted not just by coughing and sneezing but also by speaking^10^) quickly turn into aerosols (smaller microdroplets),^11^ so unless they are controlled at source, they become much harder to block.

A crucially important point, which is often overlooked by doctors, systematic reviewers and the lay press (and which was not addressed at all by Martin et al), is that most research on masks and face coverings—almost all of which has been undertaken in the context of health care workers—considers the extent to which they *protect the wearer*. The current question we need to address is a different one: whether covering the face *protects other people from droplets emitted by the wearer*—a measure known as source control. Source control works in a different way to wearer‐protection—by blocking large droplets as they are emitted in coughing, sneezing, and talking and before they become aerosolised.^10^, ^12^, ^13^, ^14^ Large droplets (and indeed a proportion of aerosols) are blocked—not perfectly, but significantly—by cotton home‐made coverings.^10^, ^15^, ^16^, ^17^, ^18^

Face coverings that protect the wearer work by blocking tiny aerosolised particles. For this reason, medical‐grade masks need to meet stringent filtration standards, about which much has been written (see for example^19^). In contrast, source control face coverings can potentially be very effective even if they only block the larger droplet particles. Studies of the efficacy of masks in protecting the wearer are therefore irrelevant to the question of source control.

Evidence of asymptomatic carriage of Sars‐CoV‐2 is strong and consistent. Oran and Topol have analysed (to date) 12 such examples from around the world,^20^ including cohorts identified for nationwide testing (Iceland), local population testing (Vo, Italy), passengers or crew of three ships (Diamond Princess, USS Theodore Roosevelt, and Charles de Gaulle aircraft carrier), nursing home staff and residents (United States), residents of two homeless shelters (Boston and Los Angeles), ex‐pats (Japanese evacuated from Wuhan and Greek citizens evacuated from other countries), and pregnant women (New York City obstetric patients). In these diverse cohorts, between 31% and 88% of positive cases were asymptomatic or pre‐symptomatic when tested. A recent editorial in the New England Journal of Medicine argued that the exceptionally high rates of asymptomatic transmission of Sars‐CoV‐2 call for a different approach to infection control—specifically, masks or face coverings for the public.^21^

In contrast to the high transmission rates from such individuals in this case series, there are some impressive case examples of infected individuals *not* passing on the virus when wearing a mask. For example one man flew from China to Toronto wearing a mask for the entire flight, became symptomatic the next day and tested positive for covid‐19; none of the other passengers or crew became infected.^22^

Another piece of evidence that covering the face could make a big difference is super‐spreader events, a list of which has been compiled by Kay.^20^ Perhaps the most dramatic is the choir practice in Seattle, in which, despite maintaining a degree of social distancing during the rehearsal, 45 of 60 people became infected and two (so far) have died.^23^ In all these super‐spreader events, extensive transmission was traced back to close contact—but not necessarily physical touching. As the authors put it: “*When do COVID‐19 [super‐spreader events] happen? … Wherever and whenever people are up in each other's faces, laughing, shouting, cheering, sobbing, singing, greeting, and praying*.”

In relation to a community‐wide intervention such as face coverings, we do not need to prevent every transmission of every droplet or every viral particle. As with hand‐washing and social distancing, the objective of the policy is more modest: to achieve a *substantial reduction* in the transmission rate of the virus. Every infectious disease has a transmission rate (R0). A disease with an R0 of 1.0 means that each infected person, on average, infects one other person. A disease whose R0 is less than 1.0 will die out. The strain of flu that caused the 1918 pandemic had an R0 of 1.8. The R0 of Sars‐CoV‐2 was estimated at 2.4 by Imperial College researchers,^24^ and other research suggests it could even higher.^25^ A population measure that reduces the transmission rate (“effective R0” or R_eff_) to below 1.0 will be highly effective, *even if some cases of transmission still occur*.

Mathematical modelling suggests that a face covering that is 60% effective at blocking viral transmission and is worn by 60% of the population will reduce R0 to below 1.0.^26^ This leaves plenty of room for error as people make their own imperfect coverings from old clothing and as some people either cannot or will not wear a face covering. Not all respiratory viruses are filtered equally; masks appear to be more efficient at blocking Sars‐Cov‐2 than rhinoviruses or adenoviruses, for example.^27^ Materials scientists have shown that whilst different fabrics are more or less efficient at blocking particle transmission, cotton weaves with high thread count or a double layer of two different fabrics (eg, cotton‐flannel) typically provides high filtration efficiency.^28^

There are now many natural experiments of the wearing of masks or face coverings in Covid‐19, as countries introduce either mandatory or voluntary policies. Of note is the example of the Czech Republic and Austria, both of which introduced social distancing on the same day; the former also introduced compulsory face coverings. New covid‐19 infections fell more quickly in the Czech Republic, and only began to fall in Austria after masks were made mandatory 2 weeks later,^3^ though an alternative interpretation of this natural experiment is that Austrian data was confounded by changes in testing policy.^29^ Also noteworthy is the observation that every single country where masks or cloth face coverings have been introduced as national policy (often but not always alongside other measures), rates of transmission fell in the subsequent days.

All these various streams of evidence contribute, in different ways and at different levels, to strengthen the argument for recommending face coverings, especially in crowded public places where social distancing is impossible, during the pandemic. With this wider evidentiary context sketched, let me now take on the specific claims made by Martin et al in their paper and rapid response.^4^, ^5^

## 3 PRECAUTIONARY PRINCIPLE, “WEAK” EVIDENCE AND POTENTIAL HARMS

*The precautionary principle we invoked to justify wearing of masks* ^1^ *is, Martin et al imply, irrelevant, because it is normally used to advise caution in the uptake of innovations with known benefits but uncertain or unmeasurable downsides, such as exposure of the public to radiation*.

The term “precautionary principle” does not have a fixed meaning, though I accept that it is more usually invoked as described by Martin et al. It may surely prove equally appropriate (a) when harm is not currently happening but a proposed intervention may cause harm and (b) when serious harm is currently happening and a proposed intervention may reduce that harm. There seems to me to be a strong symmetry between these examples. One does not cancel the other out. Both the omission in the former case and the act in the latter case are measures aimed at preventing harm.

*“[T]he very weak evidence for face masks should be reiterated”. Trials, say Martin et al, have shown no evidence of reduced transmission with masks compared to no masks, and observational studies are contaminated with multiple confounders (e.g. parallel introduction of other measures such as hand washing)*.

The evidence base for face coverings (described above) is not weak. However, it was a weak rhetorical move for Martin et al to ignore the strongest evidence when penning their critique. Our BMJ analysis article briefly reviewed the literature from experimental trials and systematic reviews.^1^ Two preprint systematic reviews^30^, ^31^ and a narrative review^6^ were all published the same week. In all those syntheses, there is a conspicuous *absence of experimental evidence* in relation to the wearing of masks *in public places, by the lay public*, as ***source control*** to *prevent community transmission* of any respiratory illness.

The sum total of randomized trials and observational studies covered in these reviews, *all of which are irrelevant to the question of source control*, comprise: (a) studies of mask‐wearing within the home to reduce contagion to other family members^32^, ^33^, ^34^, ^35^, ^36^, ^37^, ^38^, ^39^, ^40^, ^41^; (b) studies of occupational exposure (eg, workers in poultry factories)^42^, ^43^; (c) studies of specific mass events (notably, pilgrimages to the Hajj)^44^, ^45^, ^46^, ^47^, ^48^, ^49^, ^50^; (d) studies in schools and university halls of residence^51^, ^52^, ^53^, ^54^; (e) studies of air travel^55^; (f) studies of health care workers^40^, ^56^, ^57^, ^58^; and just two studies of general community prevention: an attempt to prevent the common cold in Finland,^59^ and a paper on behavioural measures (among other things) in the prevention of SARS, in which those who “always” wore a mask when outside the home had a relative risk of developing the disease of 0.3 compared to those who “never” wore one.^60^

Almost all these primary studies were designed to test the hypothesis that wearing a mask in the specific situation described in (a) to (g) above *protects the wearer*; one or two considered the specific question of whether mask‐wearing by a sick family member protects others in the household.^35^, ^36^, ^41^ The question my colleagues and I have addressed in our articles^1^, ^2^, ^3^ was a completely different one: whether a face covering worn by a member of the general public *protects others in the community*. Martin et al's depiction of the evidence from trials and observational cohort studies as “very weak” is incorrect. Such randomized controlled trial evidence, in relation to source control, is *entirely absent* and unrelated evidence should not be presented as a possible answer (Note: this does not mean there is no evidence at all—merely, that there is no evidence valued by the RCT community).

Absence of trial evidence is partly due to the fact that experimental studies of mass public health measures are usually impractical. We do not randomize schools to close, towns to go into lockdown, people to sneeze into their elbows or whole communities to wash their hands regularly. That is simply not how mass public health interventions get tested.^61^ The argument that we should not recommend face coverings *because there are no published experiments* is out of step with other public health policy on infection control in general and covid‐19 in particular. As with other public health measures, we should make a decision based on an assessment of the full body of evidence described above.

*Wearing face masks may cause harm, say Martin et al, specifically (citing the Jefferson systematic review* ^3^ *) “discomfort, dehydration, facial dermatitis, distress, headaches, exhaustion.”*

It is widely reported that prolonged use of personal protective equipment by health care personnel in pandemic contexts is associated with all the problems listed (though exhaustion in particular may have other explanations in such circumstances). Some research studies have confirmed that prolonged wearing of medical‐grade masks by health care workers can result in physical and psychological harms.^62^, ^63^, ^64^, ^65^ However, neither Martin et al nor the Jefferson systematic review which they cite offer any evidence whatsoever that the use of home‐made face coverings by the lay public for source control has been shown to cause such harms. Indeed, there is no common sense reason why a covering made out of one's own old t‐shirt would cause illness when the t‐shirt itself was well‐tolerated (and if it wasn't, why make a mask out of it?). The possible harmful effects of face coverings (eg, anecdotal accounts of irritation behind the ears from ill‐fitting elastic) should also be weighed against their potential benefits, and the potential advantages of novel equipment such as face screens (clear plastic visors) urgently researched.^66^

## 4 CAN THE GENERAL PUBLIC BE TRUSTED?

*The general public, propose Martin et al, are unlikely to use masks “properly”. Even healthcare workers struggle to achieve necessary standards of donning and doffing technique, and “inappropriately discarded masks present an infection risk”*.

Infection control standards designed for health care workers are not directly relevant to the general public. The infected particles on a health care worker's mask are likely to come from patients, and in this situation the health care worker is (hopefully) uninfected and therefore vulnerable. In contrast, if a member of the public is wearing a cloth face covering, they are the most likely source of any infectious particles on it. The more infectious particles that are caught in that covering, the fewer will have been aerosolised to infect others. A face covering that has been removed does not need to be disinfected, and formal doffing is not needed (though hand‐washing would be sensible in case the covering has become contaminated with droplets from others). Sars‐CoV‐2 has a lipid membrane which is destroyed by soap or detergent (this, of course, is why hand‐washing works). A cloth face covering can be laundered along with other clothing in a normal hot wash.^67^ An alternative option in low‐income countries is to wash the covering with soap and water and leave it to dry in the sun. Imposing unnecessarily high standards of disinfection on the public is likely to reduce uptake of the measure and be counterproductive.

*Being able to make, don, doff and disinfect your own cloth mask, suggest Martin et al, is a middle‐class privilege. The efficacy of masks in the general population will be reduced by “the potential for great variation in materials, fit, adherence, touching and adjustment, doffing, disposal, frequency of laundering and so on”*.

There is no need to standardize the design of masks or fetishise how they are worn, any more than we do so for the shoes that protect our feet or the t‐shirts we pull over our faces. Cotton and similar materials do not block droplets entirely—but most double‐layer fabrics seem to filter up to 90% of them, especially if two different fabrics with different physical and electrostatic properties are used.^15^, ^16^, ^17^, ^18^ There is some evidence that the Sars‐CoV‐2 virus relies more heavily than influenza, adenoviruses, or rhinoviruses on droplets, and will thus be more easily filtered out by a cloth cover at source than other respiratory viruses.^68^, ^69^ As noted above, if 60% of people wear a mask that is 60% effective, this is likely to be sufficient to substantially reduce the transmission of Sars‐CoV‐2. To say that because some people may find it difficult to obtain or launder a mask or face cover, we should not recommend them for anyone is illogical—especially since adverse socioeconomic circumstances is a risk factor for developing Covid‐19 and also for poorer prognosis.^70^ The negative, individualist emphasis of Martin et al's critique ignores the positive impact of collectively making face coverings as a component of wider community resilience strategies in Covid‐19.^71^, ^72^ The South African Government, for example, has recently issued a tender for community sewing cooperatives to supply cloth face masks.^73^

*Risk compensation (in which people made to wear masks reduced other infection control behaviours such as hand‐washing), suggest Martin et al, could occur*.

My critics cite a review from 20 years ago which describes mixed findings on risk compensation behaviours.^74^ They do not cite a more recent review which suggests that such behaviours appear rare.^75^ Both these reviews, however, focused mainly on injury prevention, not on infection control measures. More relevant perhaps are studies showing that teenagers vaccinated against human papilloma virus do not appear to take more sexual risks,^76^, ^77^ though there is some evidence that pre‐exposure prophylaxis for HIV may increase sexual risk‐taking in men who have sex with men.^78^ The argument that risk compensation behaviour would occur specifically in relation to face coverings in the context of Covid‐19 is entirely speculative. It is also unlikely. If adverse behaviour change happens to a significant degree, we would surely have seen some examples from around the world by now, as numerous countries have now made the wearing of masks or face coverings mandatory. Two recent studies from Hong Kong, based on self‐reports in online or telephone surveys (hence, relatively weak evidence), found that those who reported wearing masks in public places were also more likely to report more hand‐washing and social distancing.^79^, ^80^

## 5 UNINTENDED CONSEQUENCES?

*[U]niversal mask‐wearing might aggravate the climate of fear already documented for Covid‐19*.

Fear is perhaps a reasonable response to a deadly pandemic that has so far affected at least three million people and cost hundreds of thousands of lives. There is no evidence that policies which encourage or mandate covering the face increase fear. The counterargument—that such a measure would help *reduce* fear—is equally plausible (though there is no definitive evidence either way). In studies of community mask use in tuberculosis control, mask‐wearing by affected individuals reduced disease transmission but increased stigma,^81^, ^82^ whereas promotion of mask‐wearing by all members of the community was associated with destigmatisation.^82^ The relevance of these findings to the current pandemic are unclear.

*Promoting mask‐wearing by the lay public could lead to a shortage of medical‐grade masks*.

This is a real concern, but it is not a reason to distort or deny the evidence of benefit. There is no reason why the public should wear medical‐grade masks, since cotton face coverings are more comfortable, recyclable, and sufficiently effective for source control, especially given the evidence on how this particular virus behaves (it sits in the upper respiratory tract and is emitted mostly in droplets).^68^, ^69^ A public information campaign would be needed to get this message across to lay people as well as to clinicians and scientists (most of whom, like Martin et al, have unjustifiable extrapolated findings from research on infection control in health care settings and sought to apply the same standards to the public). In any case, simple surgical masks could surely be produced easily and in large numbers by repurposing manufacturing capacity if the political will and logistical capability was there.^83^

*[B]usinesses or states might see widespread or mandatory mask‐wearing as a warrant for a premature return to ‘business as usual,’ justifying unsafe workplaces or crowded commuting conditions in terms of the protection offered by masks*.^4^

This statement is entirely speculative. No evidence is given for it and it implies that the preferred state is for society to remain in lockdown indefinitely.^84^ The risks to the economy of prolonged lockdown are dire.^85^, ^86^ Recession and job losses will have a disproportionate effect on the poor and socially excluded. There are ethical as well as scientific arguments for considering all measures that may help to reduce the lockdown period and get businesses up and running as a matter of urgency.

*Masks, suggest Martin et al, are an example of a complex intervention in a complex system. Their effects are impossible to predict, therefore we should not introduce them*.

The papers cited to support this assertion (one of which was coauthored by me^87^) actually support the opposite conclusion. Just because a complex system is unpredictable does not mean we should do nothing.^88^ As Martin et al acknowledge, careful data collection and frequent, timely analysis that feeds into adjustment of policy will allow an adaptive and data‐driven response. Their depiction of current United Kingdom policy as too “blunt” to respond in this way is conflating politics with science. It is not a legitimate reason to sit idle when hundreds are dying daily.

## 6 “SYSTEMATIC” VS NARRATIVE REVIEWS

In the first paragraph of their paper, Martin et al contrast “two [preprint] systematic reviews” with “another preprint review, with more opaque methods but encompassing an eclectic range of disciplinary perspectives.” The implication is that the conclusions of “systematic” reviews which favour controlled experiments are necessarily more reliable than those of “opaque” and “eclectic” narrative reviews which bring in so‐called anecdotal evidence and findings from basic and social sciences. Elsewhere, colleagues and I have challenged this conceptual bias.^89^ In that paper, we distinguish between narrowly defined biomedical questions that can be answered using conventional systematic review, with meta‐analysis where appropriate, and more complex, multifaceted problems that require *clarification and insight*, for which a more interpretive and discursive synthesis of is needed.

Looking back at the first part of this article, where I summarized the evidence that Martin et al chose to ignore, I am struck by the *stories* they did not examine (the Covid‐stricken choir, the air passenger whose mask may have saved a planeload of people from contagion, the cruise ships that became floating quarantine prisons). But these stories are crucial to both our scientific understanding and our moral imagination. Their contrasting plots—tragedy, melodrama, lucky escape—pull together complex chains of influence and remind us that causality in a pandemic is rarely linear. Anecdotes may be a low form of evidence in some taxonomies, but each one calls for an explanation.

As my coauthors and I concluded in out article on narrative review:

*Training in systematic reviews has produced a generation of scholars who are skilled in the technical tasks of searching, sorting, checking against inclusion criteria, tabulating extracted data and generating ‘grand means’ and confidence intervals. These skills are important, but … critics may incorrectly assume that they override and make redundant the generation of understanding. … While there are occasions when systematic review is the ideal approach to answering specific forms of questions, the absence of thoughtful, interpretive critical reflection can render such products hollow, misleading and potentially harmful*.^89^

## 7 CONCLUSION

In conclusion, I congratulate Martin et al for rising to my challenge to produce a critique of my publications on masks and face coverings for the public. But whilst academic sparring has an important place in keeping us on our toes, we also need to remember our moral accountability to a society in crisis. The relentless, day on day stories of avoidable deaths from this dreadful disease sicken me. I will do whatever I can, as an academic, a doctor and a citizen, to reduce that death toll and help get society back running again.

As Gandhi et al concluded in their NEJM editorial: “This unprecedented pandemic calls for unprecedented measures to achieve its ultimate defeat.”^21^ It is time to put the straw men to rest and embrace the full range of evidence in the context of the perilous threat the world is now facing.
